# Assessing bone mineral density in children and adolescents living with HIV and on treatment with tenofovir disoproxil fumarate: a systematic review

**DOI:** 10.1590/1984-0462/2024/42/2023042

**Published:** 2023-11-10

**Authors:** Maria Brennda Ferreira de Gusmão, Vinícius Vital de Oliveira, Natália Maria da Silva Santos, Luciana Costa Melo

**Affiliations:** a Universidade Federal de Alagoas Maceió AL Brasil Universidade Federal de Alagoas, Maceió, AL, Brasil.

**Keywords:** Child, Adolescent, HIV, Bone density, Tenofovir, Criança, Adolescente, HIV, Densidade óssea, Tenofovir

## Abstract

**Objective:**

To investigate the impact of tenofovir disoproxil fumarate on bone mineral density and bone mineral content in children and adolescents infected with the human immunodeficiency virus.

**Data source:**

The search procedure was performed according to the Preferred Reporting Items for Systematic Reviews and Meta-Analyses Statement. The search was carried out until April 2022 in Medical Literature Analysis and Retrieval System Online (Medline), Embase, Cochrane Central, Latin American and Caribbean Health Sciences Literature, Web of Science, Scopus, and MedRxiv. The combination of terms used was: (Children OR Youth OR Teenagers) AND HIV AND (Tenofovir OR “Antiretroviral therapy”) AND (“Bone density” OR Osteoporosis OR Osteopenia). The protocol was registered in the International Prospective Register of Systematic Reviews (PROSPERO, CRD42022312851)

**Data synthesis:**

The initial searches resulted in 1156 papers. After the exclusion of duplicate studies, three blinded reviewers analyzed the title and abstract of 563 papers, of which 57 remained to be read in full. Only nine papers met the eligibility criteria and were included in descriptive and risk-of-bias analyses. Regarding study design, four were cross-sectional, three were longitudinal before-after studies without a control group, and two were prospective cohorts. Among these nine papers, seven showed no significant association between tenofovir disoproxil fumarate use and reduced bone mass in young people. However, these papers did not have high methodological quality.

**Conclusions:**

Although most of the selected papers found no harmful effect of tenofovir disoproxil fumarate on bone mass, further primary research with higher methodological quality is needed so robust scientific evidences can be obtained.

## RESUMO

**Objetivo:**

Investigar o impacto do tenofovir disoproxil fumarato sobre a densidade mineral óssea e o conteúdo mineral ósseo em crianças e adolescentes infectados pelo vírus da imunodeficiência humana.

**Fontes de dados:**

O procedimento de busca foi executado de acordo com o Preferred Reporting Items for Systematic Reviews and Meta-Analyses (PRISMA) Statement. A busca foi realizada até abril de 2022 nas seguintes bases de dados: Medical Literature Analysis and Retrieval System Online (Medline), Embase, Cochrane Central, Literatura Latino-Americana e do Caribe em Ciências da Saúde (Lilacs), Web of Science, Scopus, and MedRxiv. A combinação de termos utilizada foi: (Children OR Youth OR Teenagers) AND HIV AND (Tenofovir OR “Antiretroviral therapy”) AND (“Bone density” OR Osteoporosis OR Osteopenia). O protocolo foi registrado na base International Prospective Register of Systematic Reviews — PROSPERO (CRD42022312851).

**Síntese dos dados:**

As pesquisas iniciais resultaram em 1.156 artigos. Após a exclusão dos estudos duplicados, três revisores cegos analisaram título e resumo de 563 estudos, dos quais 57 permaneceram para leitura na íntegra. Somente nove artigos atenderam aos critérios de elegibilidade e foram incluídos para análises descritivas e de risco de viés. Com relação ao desenho dos estudos, quatro foram transversais, três foram estudos longitudinais antes-depois sem grupo controle e dois foram coortes prospectivas. Dos nove artigos, sete não mostraram associação significativa entre uso de TDF e redução de massa óssea em pessoas jovens. Entretanto, esses estudos não tiveram alta qualidade metodológica.

**Conclusões:**

Embora a maioria dos estudos selecionados não tenha encontrado efeito prejudicial do TDF sobre massa óssea, novas pesquisas primárias com maior qualidade metodológica são necessárias para que sejam obtidas evidências científicas robustas.

## INTRODUCTION

The human immunodeficiency virus (HIV) infection is an epidemic disease among children and adolescents, and around 2.8 million people infected are in this age group (0–19 years).^1^ In 2020, an estimated 300,000 new children and adolescents acquired HIV worldwide.^1^

Tenofovir disoproxil fumarate (TDF) is one of the drugs used in antiretroviral therapy (ART). The Food and Drug Administration (FDA) approved its use in children and adolescents from two years of age. In Brazil, however, it is only part of the preferred therapy regimen for youth over 12 years of age.^2,3^ For children from 3–12 years old, it is indicated as part of alternative therapy.^2,3^

In this age group, bone development is crucial, because more than 90% of the bone mass is obtained till the age of 20.^4^ During infancy, bone mineral deposition occurs slowly.^5^ From puberty and growth spurt, the rate of bone formation becomes fast until it reaches a peak, which will constitute a reserve of bone quantity and quality.^5-7^ The peak of bone mineral density (BMD) occurs around age 25; however, it can vary according to the skeleton site.^5,8,9^

For every 10% increase in bone mass in adolescence, the risk of fractures is reduced by 50% and the development of osteoporosis is reduced by up to 13 years.^8^ Therefore, people who come into adulthood with low bone mass will have a higher risk of bone fracture.^10^ A lower peak of BMD is a problem, especially in girls, because pregnancy and lactation periods mobilize bone calcium to meet fetal growth needs and produce the mother’s breast milk, which predisposes to a higher risk of osteoporosis.^11^ Furthermore, women face a drop in estrogenic hormones after menopause causing higher rates of bone reabsorption.^9^ Although in men the BMD loss starts later than in women, the gain in bone mass during their puberty is also a protective factor against osteoporosis — a disease with a high prevalence in both genders.^6^

Among HIV-infected individuals, the causes of low bone mass are multifactorial, involving smoking, physical inactivity, inadequate diet, the infection itself, chronic activation of the immune system, and adverse effects of antiretroviral therapy.^12^ The virus promotes a proinflammatory condition that is not completely reversed by ART and causes increased reabsorption relative to bone formation.^12,13^ Furthermore, in vitro studies showed that proteins present in HIV stimulate osteoclastic activity and suppress osteoblastic activity.^14-16^

The TDF is a tenofovir prodrug from the nucleoside reverse transcriptase inhibitor class, which inhibits HIV proliferation.^17^ In murine models, it can decrease extracellular adenosine concentration, which increases osteoclast differentiation and causes osteopenia.^17^ Furthermore, TDF is thought to be associated with bone mass loss due to its toxicity in the proximal renal tubule with consequent phosphate loss.^18^ Clinical studies in adults have associated its use with reduced BMD.^19,20^

Because of TDF potential effect of downregulating bone turnover, it is hypothesized that there is an association between its use and reduced bone mass. However, in children and adolescents, the effect of TDF on bone mass and its clinical significance are still controversial.^14,18,21^ In this context, a systematic review is necessary to analyze the scientific evidences available in the literature and to support future research on this topic. Therefore, the present paper aimed to answer the following research question: what is the impact of TDF on bone mineral content (BMC) and BMD in children and adolescents living with HIV? 

## METHOD

This paper is a systematic literature review, outlined according to the recommendations of the Preferred Reporting Items for Systematic Reviews and Meta-Analyses (PRISMA).^22^ Prior to the systematic review development, a protocol was registered in the International Prospective Register of Systematic Reviews (PROSPERO), record: CRD42022312851, available at: https://www.crd.york.ac.uk/prospero/display_record.php?ID=CRD42022312851.

Longitudinal-observational studies, clinical trials, and cross-sectional studies that examined the effects of ART containing TDF on measures of bone mass in children and adolescents living with HIV were included in the eligibility criteria. Adolescents are considered persons aged 10–19 years, as defined by the World Health Organization.^23^ The bone mass measures considered for inclusion in the present study were BMD and BMC, both represented mainly in Z-score and measured by dual-energy X-ray absorptiometry (DXA). Exclusion criteria were: studies available only in the form of conference abstracts, review studies, case reports, studies that included children and adolescents with HIV and hepatitis B virus coinfection, patients with previous renal, bone, or endocrine diseases, use of vitamin D or non-antiretroviral drugs that affect bone metabolism. There were no exclusions regarding the language or period of the studies.

The searches for the studies to be reviewed were performed in the following online databases: Medline (in PubMed, 1966–2022), Embase (in ELSEVIER, 1947–2022), Cochrane Central (Central, until 2022), Lilacs (in Virtual Health Library – VHL, 1982–2022), Web of Science (in Clarivate, 1945–2018), and Scopus (in ELSEVIER, 1996–2018). To find gray literature, the MedRxiv database (up to 2022) was used. The last searches in the databases were performed on April 5, 2022.

The strategies for searches are available with the correspondent author and were elaborated with terms combined with Boolean operators, as follows: (Children OR Youth OR Teenagers) AND HIV AND (Tenofovir OR “Antiretroviral therapy”) AND (“Bone density” OR Osteoporosis OR Osteopenia). These terms were adapted according to the availability of exact descriptors in each controlled vocabulary platform, with Emtree being used for Embase and Scopus, and Health Science Descriptors/Medical Subject Headings (DeCS/MESH) for the other databases.

A manual search was proceeded on the reference list of the included studies to look for potential papers that could be considered for inclusion in the analysis.

The searches and the entire study selection process were performed by three independent blinded reviewers. The initial triage was conducted in Rayyan – Intelligent Systematic Review Platform. First, the platform detected duplicate papers, which were checked by one of the reviewers to exclude studies that were indeed duplicates. Subsequently, the three reviewers performed initial triage by reading the title and abstract on the platform. Eligible papers were selected by reading the full text. In cases of disagreement, the final decision was reached by consensus after discussion among the three reviewers.

To check the reliability among the reviewers, the R program^24^ was used to perform Fleiss’ kappa test, which is an adaptation of Cohen’s kappa used when there are three or more reviewers.^25,26^ For this, each reviewer’s decisions before the consensus meetings were considered. The level of reliability was interpreted according to the Kappa (k) values as follows: none (0–0.20); minimal (0.21–0.39); weak (0.40–0.59); moderate (0.60–0.79); strong (0.80–0.90); and near perfect (above 0.90).^26^ 

In the screening stage by title and abstract, Fleiss’ Kappa test presented moderate reliability among the three reviewers (k=0.696; 95%CI 0.648–0.743; z=28.6; p<0.001), with 92% agreement between inter-raters. In full-text selection, Fleiss’ Kappa test showed moderate reliability (k=0.748; 95%CI 0.577–0.918; z=8.59; p<0.001), and the percent agreement was 81.8%.

The data extracted from the included studies were organized in forms previously developed, contemplating the following items: paper identification (author and year), study design, study population (characteristics, country, sample size, age, gender of volunteers, follow-up time, and nutritional status), intervention or exposure group, control group, source of information on the use of TDF, bone mass status variables, and results. Data unavailable in the papers were requested from the authors by e-mail.

For papers with a longitudinal approach that provided bone mass variables only at baseline, cross-sectional data analysis was considered. The control group composed of individuals without HIV was not considered for data extraction. Research with no control group, but that followed the TDF group over time, regardless of whether it was intervention or exposure, was described as longitudinal before-after studies.

The evaluation of bias risk was conducted by three independent reviewers who had been previously trained, and disagreements were resolved by consensus after discussion.

For cross-sectional studies, the Quality Assessment Tool for Observational Cohort and Cross-sectional Studies Scale from the National Institutes of Health (NIH)^27^ was used. For longitudinal before-after studies, the Quality Assessment Tool for Before-After (Pre-Post) Studies with no Control Group from the NIH^28^ was used. For these two scales, the classification was made according to the percentage of “yes” answers. Thus, 70 to 90% of “yes” answers would classify the study as “good”, 41 to 69% as “fair”, and up to 40% as “poor”. 

For the longitudinal approach with the control group, the Newcastle-Ottawa Quality Assessment for Cohort Studies^29^ scale was used. On this scale, classification was performed based on the total score of each study: low risk of bias (7–9 points); high risk of bias (4–6 points); and very high risk of bias (0–3 points).

A descriptive approach was used to highlight the results of the present paper. The data analysis was categorized by study approach, including:

1. Before-after longitudinal studies without a control group;
2. Longitudinal studies with a control group; and
3. Cross-sectional studies. 

In addition, meta-analysis was considered as long as the studies met the following criteria:

1. Similar paper designs;
2. Use of similar units of bone mass measurements; and
3. Presence of similar intervention and control groups between studies.

## RESULTS

**Figure 1.**
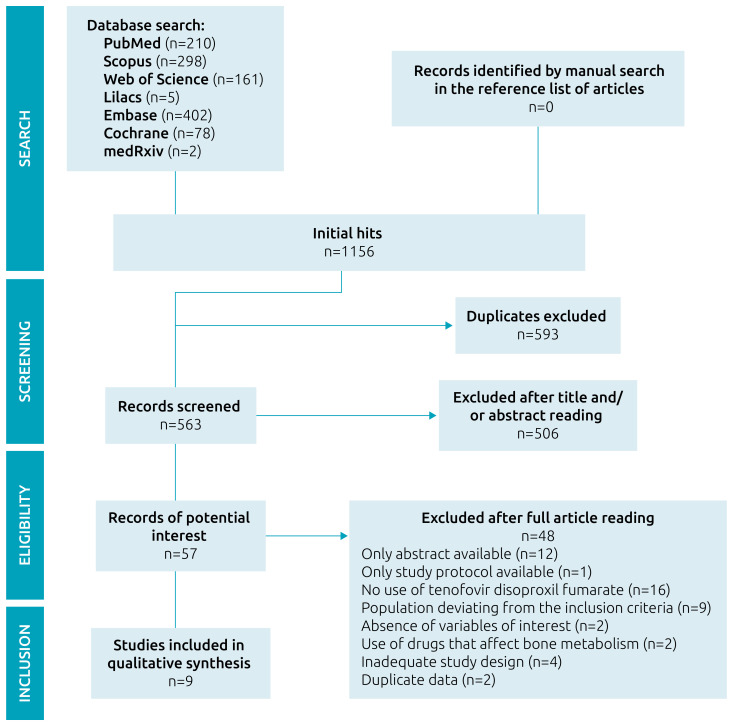
Study selection flowchart.

Database searches identified 1156 papers. After the exclusion of duplicates, 563 references were screened by title and abstract analysis. Among these, 57 met the eligibility criteria, and their full texts were analyzed. Thus, nine studies were included in this review. Finally, a manual search was performed in the references of the included studies, but no article met the inclusion criteria (Figure 1).

Among the nine included studies, two were conducted in Italy,^30,31^ one in the Netherlands,^32^ one in Canada,^33^ one in Thailand,^34^ one in Brazil and Panama,^35^ one in Thailand and Indonesia,^36^ one in Zimbabwe,^13^ and one did not inform the country of origin.^37^ Regarding the paper approach, four were cross-sectional, three were longitudinal before-after studies without a control group, and two were prospective cohort studies (Tables 1 and 2). 

**Table 1.** Characteristics of the longitudinal studies included.

Author	1. Study population 2. Sample (n)	Objectives	1. Study design 2. Statistical analysis	Outcomes*
Hazra et al.^37^	1. HIV-infected children and adolescents who had progressive disease even after using 2 antiretroviral regimens. 2. n=18.	Provide preliminary TDF safety data.	1. Longitudinal before-after. 2. Wilcoxon Test and Spearman Correlation.	The median decrease in LS BMDz was statistically significant at week 24 (p=0.007) and at week 48 (p=0.02). However, reductions in BMDz at week 48 were not correlated with TDF pharmacokinetic parameters.
Negra et al.^35^	1. Adolescents infected with HIV-1, with previous ART failure and with a viral load greater than 1000 copies/mL. 2. n=81.	To evaluate the efficacy and safety of TDF in adolescents.	1. Longitudinal before-after. 2. Wilcoxon Test and Mann-Whitney Test.	Median LS BMD increased at weeks 144 (p<0.001), 192 (p<0.001), 240 (p<0.002) from baseline. The median reduction in LS BMDz adjusted for chronological age was -0.265 (p=0.001) at week 96. However, after 288 weeks there as an increase in LS BMDz.
Giacomet et al.^30^	1. Children and adolescents vertically infected with HIV with previous long-term ART without TDF. 2. n=26.	Evaluate the effect of TDF on bone mass in young people with HIV	1. Longitudinal before-after. 2. Linear mixed effects model.	BMD changes observed over the years in HIV-infected patients did not differ from those expected in healthy children and adolescents, indicating that the TDF-containing regimen did not impair skeletal mineralization. After 12 months, mean LS BMDz increased by 0.2 in the total sample.
Bunders et al.^32^	1. Children with HIV treated at Emma Children’s Hospital between 2002 and 2010. Exposed to TDF (n=13) and controls (n=53). 2. n=66.	To determine the impact of ART on the BMD of HIV-infected children.	1. Longitudinal. 2. Univariate and multivariate linear regressions.	Despite the negative trend observed between femoral BMDz-score and TDF treatment, femoral (p=0.244) and lumbar spine (p=0.584) BMDz-scores were not significantly lower among children treated with TDF in univariate regression.
Aurpibul et al.^34^	1. HIV-infected children with viral suppression after regimen without TDF. Intervention (n=40) and controls (n=40). 2. n= 80.	Evaluate the efficacy, safety and pharmacokinetics of TDF.	1. Longitudinal with control group. 2. Analysis of variance for repeated measures.	Significant reduction in LS BMDz occurred in children receiving TDF in the first 24 weeks of treatment (p=0.011), however there were no significant changes after 96 weeks of follow-up. No correlation was seen between tenofovir and absolute change in LS BMD (p=0.086).

* The outcomes are presented according to the control for confounding variables if the study used group pairing or regression analysis. HIV: Human Immunodeficiency Virus; TDF: tenofovir disoproxil fumarate; BMDz: bone mineral density z score; ART: antiretroviral therapy; BMD: bone mineral density; LS: lumbar spine.

**Table 2.** Characteristics of the cross-sectional studies included.

Author	1. Study population 2. Sample (n)	Objectives	1. Study design 2. Statistical analysis	Outcomes*
Zuccotti et al.^31^	1. HIV-infected children and adolescents on long-term ART or naïve to ART. Exposed to TDF (n=28). 2. n=86.	To evaluate the role of different antiretroviral regimens on bone mass measurements.	1. Cross-sectional. 2. Multiple linear regression.	Only stavudine (p=0.001) and PI (p=0.03) significantly interfered in reduction of LS BMDz. There was no correlation between the use of TDF and changes in LS BMDz (p>0.05). The LS BMC values of patients using TDF did not differ from healthy or ART naïve subjects (p>0.05).
Macdonald et al.^33^	1. Children and adolescents vertically infected with HIV. Exposed to TDF (n=11). 2. n=31.	To investigate the association of bone mass measurements with the use of ART.	1. Cross-sectional. 2. Univariate regression analysis.	There was no significant association between use of TDF and changes in LS BMCz (p>0.05) and TB BMCz (p>0.05) despite the positive trend.
Sudjaritruk et al.^36^	1. Vertically HIV-infected adolescents with long-term viral suppression on ART. Exposed to TDF (n=136). 2. n=394.	Determine the effect of TDF on metabolism and bone mass.	1. Cross-sectional. 2. Mann-Whitney test and multiple linear regression.	There was no significant difference in median BMDz between TDF users and non-users (p=0.27). The use of TDF had no significant association with changes in LS BMDz (p=0.09). There was no significant relationship between BMDz and duration of TDF treatment (p=0.34).
Rukuni et al.^13^	1. Children and adolescents using ART for at least 2 years. Exposed to TDF (n=95). 2. n=279.	Identify the prevalence of low bone density and investigate associated risk factors.	1. Cross-sectional. 2. Chi-square, Fisher’s exact test; Univariate and multiple linear regressions.	TDF use was significantly associated with lower TBLH BMCz (p=0.015) and LS BMADz (p=0.012) relative to non-TDF users. TDF was associated with decreased LS-BMADz in univariate (p<0.0001) but not multivariate analysis (p=0.25). Regarding TBLH-BMCz, there was also this association in both the univariate (p=0.037) and multivariate (p=0.046) analysis. TDF use for more than 4 years was correlated with more pronounced TBLH-BMCz deficits in multivariate analysis (p=0.046).

* The outcomes are presented according to the control for confounding variables if the study used group pairing or regression analysis. HIV: Human Immunodeficiency Virus; ART: antiretroviral therapy; TDF: tenofovir disoproxil fumarate; PI: protease inhibitors; LS: lumbar spine; BMDz: bone mineral density Z-score; BMC: bone mineral content; BMCz: bone mineral content z score; BMADz: bone mineral apparent density; TBLH: total body less-head.

The selected papers included a total of 1040 individuals with HIV diagnoses.^13,30-37^ Considering the six papers that had a control group,^13,31-34,36^ 323 patients were in the TDF group while 613 were in the control group. The proportion of female participants varied from 38.7 to 61% and was unavailable in the study by Negra et al.^35^ Of the five longitudinal research,^31,33-35,37^ four reported clearly that the patients taking TDF during the follow-up period were not using the drug before the start of the research.^31,34,37^ The follow-up time varied from one year to 7.1 years between the studies. Two of these five studies included only patients with prior ART failure.^35,37^ All papers presented the BMC or BMD variables in Z-score form, which is the most suitable for analyzing bone mass in children and adolescents (Tables 1 and 2).

Regarding the concomitant use of other antiretrovirals that may interfere with bone mass, there are five, of the nine studies, with unavailable data.^13,31-33,35^ According to Sudjaritruk et al.,^36^ 58.1% of the TDF group also used protease inhibitors (PI). In other studies, PI^37^ and efavirenz^31,34^ were used in combination with TDF in all patients.

The overall methodological quality of studies included in this review ranged from low to moderate. Scores on the Newcastle-Ottawa scale were 5 and 6, indicating a high risk of bias. Bunders et al.^32^ scored 3 for participant selection, 1 for comparability, and 1 for outcome. Aurpibul et al.^34^ scored 3, 2, and 1, respectively.

On the NIH scale for longitudinal before-after studies, two of three studies had fair quality while one had poor quality (Table 3). As for the NIH scale for cross-sectional studies, there were two with fair risk and another two with poor quality (Table 4).

**Table 3.** Quality analysis before-after studies included in this review.

Author	Defined goal	Specified population	Participation rate of eligible persons equal to 100%	Representative sample	Adequate sample size	Weel-defined intervention/exposure	Reliable measurement of outcome	Blinding of evaluators	Follow-up loss ≤ 20%	Appropriate statistical analysis	Dependent variable measured multiple times before and after the intervention	Statistical analysis for intervention at the group level	Quality rating
Hazra et al.^37^	●	●	●	●	●	●	●	●	●	●	●	●	Fair
Negra et al.^35^	●	●	●	●	●	●	●	●	●	●	●	●	Fair
Giacomet et al.^30^	●	●	●	●	●	●	●	●	●	●	●	●	Poor

Label 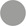 (Yes) 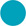 (No) 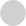 (Not reported) 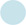 (Not applicable)

**Table 4.** Quality analysis of cross-sectional studies.

Author, year	Defined goal	Specified population	Participation rate of eligible persons >50%	Pre-specified and uniform eligibility criteria	Representative sample size	Exposure measured before the outcome	Adequate follow-up time	Weel-defined intervention/exposure	Reassessment of intervention/exposure	Weel-defined outcome	Evaluators blinding	Follow-up loss ≤ 20%	Statistical adjustment for confounding factors	Quality
Rukuni et al.^13^	●	●	●	●	●	●	●	●	●	●	●	●	●	Fair
Sudjaritruk et al.^36^	●	●	●	●	●	●	●	●	●	●	●	●	●	Poor
Zuccotti et al.^31^	●	●	●	●	●	●	●	●	●	●	●	●	●	Poor
Macdonald et al.^33^	●	●	●	●	●	●	●	●	●	●	●	●	●	Fair

Label 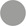 (Yes) 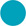 (No) 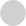 (Not reported) 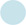 (Not applicable)

The design of this systematic review included a meta-analysis of BMC and BMD. However, upon reviewing the papers, it was impossible to perform this statistical procedure for the following reasons:

1. Different study designs;
2. Use of different bone mass measurement units;
3. Use of different measurements of central tendency and dispersion to show the data; and
4. Studies with different control or intervention groups.

## DISCUSSION

The focus of this systematic review was to identify the possible effect of TDF on bone mass in children and adolescents with HIV, in terms of BMDz (Z-score of BMD) or BMCz (Z-score of BMC) measured by DXA.

BMDz or BMCz measures of central tendency were negative at baseline in four of five longitudinal studies, indicating bone mass below that expected for the age group of subjects.^30,32,35,37^ The four cross-sectional studies followed the same tendency.^13,31,33,36^

The three longitudinal before-after studies^30,35,37^ had a total of 104 participants, who took TDF-containing regime after the previous ART and with statistical analysis suitable for before-after studies. The period between the suspension of the previous ART and the introduction of TDF was one week in Hazra et al.,^37^ the other studies did not provide such data.^30,35^ Two papers found no association between TDF and decreased bone mass.^30,35^ In contrast, Hazra et al.^37^ found median reductions in BMDz of -0.38 at week 24 (p=0.007) and -0.30 at week 48 (p=0.020) compared to a baseline median of -1.18. In this study, all volunteers’ ART contained PI, which may have influenced the decrease in BMDz, as there are reports of an association between PI and bone loss.^38,39^

In the same direction, Negra et al.^35^ observed, at week 96, a decrease in BMDz of the chronological age-adjusted spine (p=0.001), but at week 288, there was an increase without statistical significance, indicating stabilization of bone mass. Such results should be considered carefully since the loss at follow-up was 96% at the end of the study. However, the results of Negra et al.^35^ and Hazra et al.^37^ supported the information that in the first two years after initiation of various ART regimens, BMD is reduced, but stabilizes thereafter.^18^

Giacomet et al^30^ found significant increases in BMD of the lumbar spine and total body after 52 weeks of therapy containing TDF (p<0.0001). In contrast to Hazra et al.,^37^ in the Giacomet et al.^30^ study, all participants used efavirenz in combination therapy – a drug related to higher bone mass when compared to protease inhibitors^40^ — which may have influenced the increase in BMD. However, the statistical analyses were performed only for absolute BMD values, which represents a shortcoming. The BMDz and BMCz are the most accurate measures for diagnosing low bone mass in children and adolescents.^4,41,42^

Among the longitudinal researches with the control group, one had TDF as an intervention in open-label design^34^ and another as exposure.^32^ Bunders et al.^32^ demonstrated no significant association between TDF use and changes in BMDz of the lumbar spine (p=0.584) and femoral neck (p=0.244). These results are limited because of the sample size, composed of only 13 children exposed to ART. However, an association was found between longer exposure time to ART and increased BMDz of the lumbar spine (p=0.027) after a mean of 7.6 years of ART use, which corroborates the hypothesis of BMD stabilization after an initial TDF associated reduction.^18^

Aurpibul et al.^34^ found a significant decrease in BMDz of the lumbar spine only at week 24 (p=0.011) in TDF users. After 96 weeks, BMDz rose again despite not reaching the baseline value after 96 weeks of follow-up. Among the 40 children using TDF, 11 showed a significantly greater absolute reduction in BMDz of the lumbar spine than the group without TDF (p=0.002). However, the prevalence of low BMDz was 9% at the end of the study, with no significant differences between groups (p=0.395), and there was also no correlation between TDF and absolute BMD change (p=0.859).

The cross-sectional papers covered 790 patients, of whom 270 were using TDF. Three of them were adjusted for confounding factors by multivariate linear regression.^13,31,36^

Zuccotti et al.^31^ found no association of TDF use with decreased bone mass in multiple regression (p>0.050). Similarly, Macdonald et al.^33^ observed no association of TDF with BMCz values (p>0.050).

In Sudjaritruk et al.^36^ despite TDF users had lower median BMDz than nonusers, statistical significance was absent (p=0.270). In multivariate regression, no association occurred between TDF and BMDz (p=0.090). On the other vein, TDF was a predictor of an increase in bone resorption markers (p=0.040) and a decrease in bone formation markers (p=0.040). Resorption markers may indicate a high rate of bone loss and predict fracture risk independently of BMD values,^43^ and may change early about the radiopaque structure observed on DXA.^44^

Rukuni et al.^13^ showed a significant reduction in BMCz of total body less head (p=0.015) and apparent BMDz of the lumbar spine (p=0.012) in young people on TDF when compared to those on ART without TDF, in addition to associating pronounced bone deficits with longer time of exposure to TDF. These findings are consistent with Schtscherbyna et al.,^45^ in which time of TDF use was inversely correlated with BMDz of lumbar spine in a cross-sectional design. 

The bias risk evaluation showed that the papers included in this review did not have a robust methodology. Most of them do not require sample size calculation and had a small sample size, affecting the representativeness of the population.^46^ All the studies have a risk of measurement bias due to the lack of shielding of evaluators from exposure.^27,28,46^ 

In longitudinal before-after research, extensive loss to follow-up,^35^ and lack of multiple measurements of dependent variables before the intervention^30,37^ were significant methodological problems. Reliability of TDF exposure was doubtful in two studies: one had only 38.3% adherence,^35^ while another did not assess adherence.^30^

In the cross-sectional research, the bias occurred mainly due to issues inherent to the methodological design itself that prevent inference of causality:^46^ the impossibility of measuring exposure before the endpoint and follow-up time.

The evaluation of BMDz and BMCz in children and adolescents needs to consider physiological and behavioral variables that can influence these outcomes, such as menarche in girls, sexual maturation, growth spurt, diet, physical activity, solar exposure, nutritional status, ethnicity, and bone age.^4,5,6,8,42,47-51^ Furthermore, when evaluating children and adolescents with HIV, it can be viewed that there are additional confounding variables related to infection: viral load, CD4+, and therapeutic factors.^52,53^

Only Bunders et al.^32^ considered ethnicity as an independent variable in the regression analyses. This variable showed no effect on BMDz of the lumbar spine but there was a positive association between black and mixed black ethnicities and BMDz of the femoral neck.

The importance of assessing nutritional status occurs because both obesity and malnutrition are relevant factors for the reduction of BMD in children.^51^ Only one of the nine studies did not present height and weight Z-scores.^32^ In studies with available data,^13,30,31,33-37^ the mean or median of these measurements at baseline were negative, indicating that young people with HIV were shorter and lighter than healthy people, but the mean and/or median were within the limits considered for eutrophic individuals. Only Rukuni et al.^13^ reported the presence of 26% of the sample with malnutrition. Despite the importance of nutritional status in the assessment of BMD, the studies did not present a statistical analysis of the association between these variables, which may configure a fragility of the results.

No study considered nutritional status and bone age as confounding factors. Among the longitudinal before-after studies^30,35,37^ that compose the present review, none analyzed other variables with the potential to interfere with bone mass, which limits the power of their findings.

Among the longitudinal papers with the control group, one performed adjustments for HIV-related confounding factors such as percentage of CD4 cells, viral load, and duration of ART by multivariate regression,^32^ while another adjusted for age, sex, and CD4 cell count by group matching.^34^ In the study with the control group by Bunders et al.,^32^ 23% of TDF users were already using the drug before the start of the study, which makes it impossible to conclude if TDF-related bone mass changes were absent at the beginning of the research.

Regarding the follow-up time, the longitudinal studies ranged from 48 weeks to 7.1 years and only two lasted more than two years,^32,35^ representing a limitation in determining the real effect of chronic exposure to TDF on bone mass.

Of the cross-sectional research, only Macdonald et al.^33^ did not control for confounding variables. Other papers^13,31,33^ did not cover the following factors: viral load,^31,36^ CD4 count,^31^ duration of ART,^13,31^ current ART regimen,^13,31^ menarche,^13,31,36^ growth spurt,^13,31,36^ physical activity,^31^ diet,^31^ solar exposure,^13,31^ ethnicity,^13,31,36^ nutritional status,^13,31,36^ and bone age.^13,31,36^

Zuccotti et al.,^31^ removed sexual maturity from its final analyses since it was not statistically significant. However, Arabi et al.^54^ noted that BMD of lumbar spine was 43% higher in post-pubertal boys than in prepubertal boys, and 66% higher in post-pubertal girls (p<0.001). Burns et al.^55^ recommend considering Tanner stage in the evaluation of BMDz because pubertal delay influences bone mass and prevents the use of BMDz as a diagnostic criterion for osteoporosis.

The study by Sudjaritruk et al.^36^ showed that there were no differences in solar exposure, dietary calcium ingestion, and physical activity level between groups, thus, multivariate analyses to control for these factors were not necessary. Factors like age, Tanner stage, use of other antiretroviral drugs, and duration of ART were excluded from regression analysis because they showed statistical collinearity with TDF administration, which precludes conclusions about the impact of these variables on bone mass.

Rukuni et al.^13^ controlled for the largest number of confounding factors in multivariate regression. After such adjustments, only early stages of Tanner (p=0.029) and, in contradiction to Hazra et al.,^37^ higher age (p=0.0012) were significant covariables for low BMDz of the lumbar spine. In Rukuni et al. study,^13^ measures of the central tendency of bone mass were negative at all pubertal stages but did not reach low bone mass values. The other studies did not provide this data.

Furthermore, other antiretroviral drugs, such as efavirenz and PI, are known to have potential to affect BMD.^4,38,39,40^ As combinations of antiretroviral agents used in ART vary across research and among participants themselves in the same research, the observed effects on bone mass may not be exclusively attributable to TDF.

Seven of the nine studies reviewed in the present paper found no significant association between TDF and reduced BMD, but these results should be interpreted cautiously because of methodological limitations and lack of evaluation of potential confounding factors, which prevent conclusions about the real effect of TDF on bone mass.

The evidence of an association between TDF and bone mass has limitations due to the methodological designs of the papers included in this review. Randomized clinical research was not found. There was only one clinical trial with control and intervention group. Most studies were cross-sectional or longitudinal observational. Some of the papers did not show sample size calculations to ensure that the sample was representative of the general population. The studies did not use the same confounding factors in the multivariate regression analyses. Besides, the number of confounding factors was limited in most studies. Therefore, it is recommended that further research be conducted, including a sample size calculation, blinding the evaluators to exposure or intervention, and considering confounding factors in experimental design and data analysis.

The academic evidence available until now is insufficient to evidence the effect of TDF on bone mass in children and adolescents living with HIV. Although most available data indicate no association between the variables analyzed by the studies included in this review, the extant literature presents low or moderate methodological quality. Therefore, we recommend that studies of high methodological quality be conducted to identify the real effect of antiretroviral therapy containing TDF on bone health in this population.

## References

[1] 1. United States of America. United Nations Plaza New York [homepage on the Internet]. 2021 HIV and AIDS global snapshot: pregnant women, children and adolescents [cited 2022 Jul 01]. Available from: https://www.childrenandaids.org/sites/default/files/2022-01/211209_HIV%20Global%20Snapshot_V15_0.pdf

[2] 2. Brazil. Ministério da Saúde. Secretaria de Vigilância em Saúde. Departamento de Vigilância, Prevenção e Controle das Infecções Sexualmente Transmissíveis, do HIV/Aids e das Hepatites Virais. Protocolo clínico e diretrizes terapêuticas para manejo da infecção pelo hiv em crianças e adolescentes. Brasília: Ministério da Saúde; 2018.

[3] 3. United States of America. Office of AIDS Research Advisory Council (OARAC) [homepage on the Internet]. Panel on Antiretroviral Therapy and Medical Management of Children Living with HIV. Department of Health and Human Services. Guidelines for the Use of Antiretroviral Agents in Pediatric HIV Infection. United States of America: OARAC; 2022 [cited 2023 Jul 01]. Available from: https://clinicalinfo.hiv.gov/sites/default/files/guidelines/documents/pediatric-arv/guidelines-pediatric-arv.pdf

[4] 4. Sociedade Brasileira de Pediatria. Departamento Científico de Endocrinologia. Guia prático de atualização: osteoporose em crianças e adolescentes. Rio de Janeiro: Sociedade Brasileira de Pediatria; 2018.

[5] 5. Weaver CM, Gordon CM, Janz KF, Kalkwarf HJ, Lappe JM, Lewis R, et al. The National Osteoporosis Foundation’s position statement on peak bone mass development and lifestyle factors: a systematic review and implementation recommendations. Osteoporos Int. 2016;27:1281-386. https://doi.org/10.1007/s00198-015-3440-3 10.1007/s00198-015-3440-3 PMC4791473 26856587

[6] 6. Silva CC, Goldberg TB, Nga HS, Kurokawa CS, Capela RC, Teixeira AS, et al. Impact of skeletal maturation on bone metabolism biomarkers and bone mineral density in healthy Brazilian male adolescents. J Pediatr (Rio J). 2011;87:450-6. https://doi.org/10.2223/JPED.2125 10.2223/JPED.2125 22012503

[7] 7. Lima LR, Silva RC, Giuliano IC, Sakuno T, Brincas SM, Carvalho AP. Bone mass in children and adolescents infected with human immunodeficiency virus. J Pediatr (Rio J). 2012;89:91-9. https://doi.org/10.1016/j.jped.2013.02.014 10.1016/j.jped.2013.02.014 23544816

[8] 8. Lourenço B, Queiroz LB. Crescimento e desenvolvimento puberal na adolescência. Rev Med (São Paulo). 2010;89:70-5.

[9] 9. Carvalho MA, Lanna CC, Bertolo MB, Ferreira CA. Reumatologia: diagnóstico e tratamento. 5^a^ ed. Rio de Janeiro: Guanabara Koogan; 2019.

[10] 10. Heaney RP, Abrams S, Dawson-Hughes B, Looker A, Marcus R, Matkovic V, et al. Peak bone mass. Osteoporos Int. 2000;11:985-1009. https://doi.org/10.1007/s001980070020 10.1007/s001980070020 11256898

[11] 11. Winter EM, Ireland A, Butterfield NC, Haffner-Luntzer M, Horcajada MN, Veldhuis-Vlug AG, et al. Pregnancy and lactation, a challenge for the skeleton. Endocr Connect. 2020;9:R143-57. https://doi.org/10.1530/EC-20-0055 10.1530/EC-20-0055 PMC7354730 32438342

[12] 12. Puthanakit T, Siberry GK. Bone health in children and adolescents with perinatal HIV infection. J Int AIDS Society. 2013;16:18575. https://doi.org/10.7448/IAS.16.1.18575 10.7448/IAS.16.1.18575 PMC3687077 23782476

[13] 13. Rukuni R, Rehman AM, Mukwasi-Kahari C, Madanhire T, Kowo-Nyakoko F, McHugh G, et al. Effect of HIV infection on growth and bone density in peripubertal children in the era of antiretroviral therapy: a cross-sectional study in Zimbabwe. Lancet Child Adolesc Health. 2021;5:569-81. https://doi.org/10.1016/S2352-4642(21)00133-4 10.1016/S2352-4642(21)00133-4 PMC8295041 34139202

[14] 14. Fakruddin JM, Laurence J. HIV envelope gp120-mediated regulation of osteoclastogenesis via Receptor Activator of Nuclear Factor B Ligand (RANKL) secretion and its modulation by certain HIV protease inhibitors through interferon-/RANKL cross-talk. J Biol Chem. 2003;278:48251-8. https://doi.org/10.1074/jbc.M304676200 10.1074/jbc.M304676200 12975380

[15] 15. Fakruddin JM, Laurence J. HIV-1 Vpr enhances production of receptor of activated NF-κB ligand (RANKL) via potentiation of glucocorticoid receptor activity. Arch Virol. 2004;150:67-78. https://doi.org/10.1007/s00705-004-0395-7 10.1007/s00705-004-0395-7 15449141

[16] 16. Cotter EJ, Malizia AP, Chew N, Powderly WG, Doran PP. HIV proteins regulate bone marker secretion and transcription factor activity in cultured human osteoblasts with consequent potential implications for osteoblast function and development. AIDS Res Hum Retroviruses. 2007;23:1521-30. https://doi.org/10.1089/aid.2007.0112 10.1089/aid.2007.0112 18160010

[17] 17. Conesa-Buendía FM, Llamas-Granda P, Larrañaga-Vera A, Wilder T, Largo R, Herrero-Beaumont G, et al. Tenofovir causes bone loss via decreased bone formation and increased bone resorption, which can be counteracted by dipyridamole in mice. J Bone Miner Res. 2019;34:923-38. https://doi.org/10.1002/jbmr.3665 10.1002/jbmr.3665 30645771

[18] 18. Brazil. Ministério da Saúde. Secretaria de Vigilância em Saúde. Departamento de Vigilância, Prevenção e Controle das Infecções Sexualmente Transmissíveis, do HIV/Aids e das Hepatites Virais. Protocolo clínico e diretrizes terapêuticas para manejo da infecção pelo HIV em adultos. Brasília: Ministério da Saúde; 2018.

[19] 19. Baranek B, Wang S, Cheung AM, Mishra S, Tan DH. The effect of tenofovir disoproxil fumarate on bone mineral density: a systematic review and meta-analysis. Antivir Ther. 2020;25:21-32. https://doi.org/10.3851/IMP3346 10.3851/IMP3346 32077867

[20] 20. Güerri-Fernández R, Lerma-Chippirraz E, Marron AF, García-Giralt N, Villar-García J, Soldado-Folgado J, et al. Bone density, microarchitecture, and tissue quality after 1 year of treatment with tenofovir disoproxil fumarate. AIDS. 2018;32:913-20. https://doi.org/10.1097/QAD.0000000000001780 10.1097/QAD.0000000000001780 29424785

[21] 21. Aurpibul L, Puthanakit T. Review of tenofovir use in HIV-infected children. Pediatric Infect Dis J. 2015;34:383-91. https://doi.org/10.1097/INF.0000000000000571 10.1097/INF.0000000000000571 25247583

[22] 22. Page MJ, McKenzie JE, Bossuyt PM, Boutron I, Hoffmann TC, Mulrow CD, et al. The PRISMA 2020 statement: an update guideline for reporting systematic reviews. BMJ. 2021;372:n71. https://doi.org/10.1136/bmj.n71 10.1136/bmj.n71 PMC8005924 33782057

[23] 23. Brazil. Ministério da Saúde. Secretaria de Atenção à Saúde. Departamento de Ações Programáticas e Estratégicas. Proteger e cuidar da saúde de adolescentes na atenção básica. 2^a^ ed. Brasília: Ministério da Saúde; 2018

[24] 24. R Core Team [homepage on the Internet]. The R project for statistical computing. Vienna: foundation for statistical computing [cited 2022 Jul 01]. Available from: https://www.r-project.org/

[25] 25. Fleiss JL. Measuring nominal scale agreement among many raters. Psychol Bul. 1971;76:378-82. https://doi.org/10.1037/h0031619

[26] 26. McHugh ML. Interrater reliability: the kappa statistic. Biochem Med (Zagreb). 2012;22:276-82. PMID: 23092060 PMC3900052 23092060

[27] 27. National Institutes of Health [homepage on the Internet]. Quality assessment tool for observational cohort and cross-sectional studies [cited 2022 Jul 01]. Available from: https://www.nhlbi.nih.gov/health-topics/study-qualityassessment-tools

[28] 28. National Institutes of Health [homepage on the Internet]. Quality assessment tool for before after (Pre-Post) studies with no control group [cited 2022 Jul 01]. Available from: https://www.nhlbi.nih.gov/health-topics/study-quality-assessment-tools

[29] 29. Wells GA, Shea B, O’Connell D, Peterson J, Welch V, Losos M, et al. The Newcastle-Ottawa Scale (NOS) for assessing the quality of nonrandomised studies in meta-analyses. Ottawa Hospital Research Insitute; 2021.

[30] 30. Giacomet V, Maruca K, Ambrosi A, Zuccotti GV, Mora S. A 10-year follow-up of bone mineral density in HIV-infected youths receiving tenofovir disoproxil fumarate. Int J Antimicrobial Agents. 2017;50:365-70. https://doi.org/10.1016/j.ijantimicag.2017.03.026 10.1016/j.ijantimicag.2017.03.026 28689877

[31] 31. Zuccotti G, Viganò A, Gabiano C, Giacomet V, Mignone F, Stucchi S, et al. Antiretroviral therapy and bone mineral measurements in HIV-infected youths. Bone. 2010;46:1633-9. https://doi.org/10.1016/j.bone.2010.02.029 10.1016/j.bone.2010.02.029 20211284

[32] 32. Bunders MJ, Frinking O, Scherpbier HJ, van Arnhem LA, van Eck-Smit BL, Kuijpers TW, et al. Bone mineral density increases in HIV-Infected children treated with long-term combination antiretroviral therapy. Clin Infect Dis. 2013;56:583-6. https://doi.org/10.1093/cid/cis917 10.1093/cid/cis917 23097583

[33] 33. Macdonald HM, Chu J, Nettlefold L, Maan EJ, Forbes JC, Côté H, et al. Bone geometry and strength are adapted to muscle force in children and adolescents perinatally infected with HIV. J Musculoskelet Neuronal Interact. 2013;13:53-65. PMID: 23445915 23445915

[34] 34. Aurpibul L, Cressey TR, Sricharoenchai S, Wittawatmongkol O, Sirisanthana V, Phongsamart W, et al. Efficacy, safety and pharmacology of tenofovir disoproxil fumarate in virologic-suppressed HIV-infected children using weight-band dosing. Pediatr Infect Dis J. 2015;34:392-7. https://doi.org/10.1097/INF.0000000000000633 10.1097/INF.0000000000000633 25760566

[35] 35. Negra DM, Carvalho AP, Aquino MZ, Pinto JA, Silva MT, Andreatta KN, et al. Long-term efficacy and safety of tenofovir disoproxil fumarate in HIV-1-infected adolescents failing antiretroviral therapy: the final results of study GS-US-104-0321. Pediatr Infect Dis J. 2015;34:398-405. https://doi.org/10.1097/INF.0000000000000649 10.1097/INF.0000000000000649 25599284

[36] 36. Sudjaritruk T, Bunupuradah T, Aurpibul L, Kosalaraksa P, Kurniati N, Sophonphan J, et al. Impact of tenofovir disoproxil fumarate on bone metabolism and bone mass among perinatally HIV-infected Asian adolescents. Antivir Ther. 2017;22:471-9. https://doi.org/10.3851/IMP3103 10.3851/IMP3103 27786155

[37] 37. Hazra R, Gafni RI, Maldarelli F, Balis FM, Tullio AN, DeCarlo E, et al. Tenofovir disoproxil fumarate and an optimized background regimen of antiretroviral agents as salvage therapy for pediatric HIV infection. Pediatrics. 2005;116:e846-54. https://doi.org/10.1542/peds.2005-0975 10.1542/peds.2005-0975 16291735

[38] 38. Moran CA, Weitzmann MN, Ofotokun I. The protease inhibitors and HIV-associated bone loss. Curr Opin HIV AIDS. 2016;11:333-42. https://doi.org/10.1097/COH.0000000000000260 10.1097/COH.0000000000000260 PMC4838480 26918650

[39] 39. Shen Y, Shiau S, Strehlau R, Burke M, Patel F, Johnson CT, et al. Persistently lower bone mass and bone turnover among South African children living with well controlled HIV. AIDS. 2021;35:2137-47. https://doi.org/10.1097/QAD.0000000000002990 10.1097/QAD.0000000000002990 PMC8490283 34127577

[40] 40. Arpadi SM, Shiau S, Strehlau R, Patel F, Mbete N, McMahon DJ, et al. Efavirenz is associated with higher bone mass in South African children with HIV. AIDS. 2016;30:2459-67. https://doi.org/10.1097/QAD.0000000000001204 10.1097/QAD.0000000000001204 PMC5069169 27427876

[41] 41. Galindo-Zavala R, Bou-Torrent RB, Magallares-López B, Mir-Perelló C, Palmou-Fontana N, Servilla-Pérez B, et al. Expert panel consensus recommendations for diagnosis and treatment of secondary osteoporosis in children. Pediatr Rheumatol Online J. 2020;18:20. https://doi.org/10.1186/s12969-020-0411-9 10.1186/s12969-020-0411-9 PMC7041118 32093703

[42] 42. Bishop N, Arundel P, Clark E, Dimitri P, Farr J, Jones G, et al. Fracture prediction and the definition of osteoporosis in children and adolescents: the ISCD 2013 Pediatric Official Positions. J Clin Densitom. 2014;17:275-80. https://doi.org/10.1016/j.jocd.2014.01.004 10.1016/j.jocd.2014.01.004 24631254

[43] 43. Hlaning TT, Compston JE. Biochemical markers of bone turnover – uses and limitations. Ann Clin Biochem. 2014;51:189-202. https://doi.org/10.1177/0004563213515190 10.1177/0004563213515190 24399365

[44] 44. Vieira JG. Considerações sobre os marcadores bioquímicos do metabolismo ósseo e sua utilidade prática. Arq Bras Endocrinol Metab. 1999;43:415-22. https://doi.org/10.1590/S0004-27301999000600005

[45] 45. Schtscherbyna A, Pinheiro MF, Mendonça LM, Gouveia C, Luiz RR, Machado ES, et al. Factors associated with low bone mineral density in a Brazilian cohort of vertically HIV infected adolescents. Int J of Infect Dis. 2012;16:e872-8. https://doi.org/10.1016/j.ijid.2012.07.019 10.1016/j.ijid.2012.07.019 23031418

[46] 46. Hulley SB, Cummings SR, Browner WS, Grady DG, Newman TB. Delineando a pesquisa clínica. 4^a^ ed. Porto Alegre: Armed; 2015.

[47] 47. Soliman A, De Sanctis V, Elalaily R, Bedair S. Advances in pubertal growth and factors influencing it: can we increase pubertal growth? Indian J Endocrinol Metab. 2014;18(Suppl 1):S53-62. https://doi.org/10.4103/2230-8210.145075 10.4103/2230-8210.145075 PMC4266869 25538878

[48] 48. Deere K, Sayers A, Rittweger J, Tobias JH. Habitual levels of high, but not moderate or low, impact activity are positively related to hip BMD and geometry: results from a population-based study of adolescents. J Bone Miner Res. 2012;27:1887-95. https://doi.org/10.1002/jbmr.1631 10.1002/jbmr.1631 PMC3465797 22492557

[49] 49. Penner J, Ferrand RA, Richards C, Word KA, Burns JE, Gregson CL. The impact of vitamin D supplementation on musculoskeletal health outcomes in children, adolescents, and young adults living with HIV: a systematic review. PLoS One. 2018;13:e0207022. https://doi.org/10.1371/journal.pone.0207022 10.1371/journal.pone.0207022 PMC6237309 30439968

[50] 50. Kalkwarf HJ, Gilsanz V, Lappe JM, Oberfield S, Shepherd JA, Hangartner TN, et al. Tracking of bone mass and density during childhood and adolescence. J Clin Endocrinol Metab. 2010;95:1690-8. https://doi.org/10.1210/jc.2009-2319 10.1210/jc.2009-2319 PMC2853985 20194709

[51] 51. Milyani AA, Kabli YO, Al-Agha AE. The association of extreme body weight with bone mineral density in Saudi children. Ann Afr Med. 2022;21:16-20. https://doi.org/10.4103/aam.aam_58_20 10.4103/aam.aam_58_20 PMC9020628 35313399

[52] 52. Aydin OA, Karaosmanoglu HK, Karahasanoglu R, Tahmaz M, Nazlican O. Prevalence and risck factors of osteopenia/osteoporosis in Turkish HIV/AIDS patients. Braz J Infect Dis. 2013;17:707-11. https://doi.org/10.1016/j.bjid.2013.05.009 10.1016/j.bjid.2013.05.009 PMC9427423 24076108

[53] 53. Kwak MK, Lee EJ, Park JW, Park SY, Kim BJ, Kim TH, et al. CD4 T cell count is inversely associated with lumbar spine bone mass in HIV-infected men under the age of 50 years. Osteoporos Int. 2019;30:1501-10. https://doi.org/10.1007/s00198-019-04942-7 10.1007/s00198-019-04942-7 30915506

[54] 54. Arabi A, Nabulsi M, Maalouf J, Choucair M, Khalife H, Vieth R, et al. Bone mineral density by age, gender, pubertal stages, and socioeconomic status in healthy Lebanese children and adolescents. Bone. 2004;35:1169-79. https://doi.org/10.1016/j.bone.2004.06.015 10.1016/j.bone.2004.06.015 15542043

[55] 55. Burns DA, Campos Junior D, Silva LR, Borges WG. Tratado de pediatria. 4^a^ ed. Barueri: Manole; 2017.

